# A Change in the Dental Law of Ohio

**Published:** 1894-06

**Authors:** Alex. Boxwell, Andrew L. Harris

**Affiliations:** Speaker of the House of Representatives; President of the Senate


### EDITORIAL.

### A Change in the Dental Law of Ohio.

On the morning after the adjournment of the recent Legislature of Ohio, the newspapers contained the following notice:

"Mr. Lane's Dental College Bill, after much toil and tribulation, became a law in the Senate this morning. While intended to cover the special case of the Ohio Dental College, on Park street, Columbus, the bill is general in application, and will also prove of benefit to a small dental college in Cleveland."

This action of the Legislature seems to have been so hastily and so quietly done as to escape the notice of the Dental Profession, except a very few who were specially interested, or thought they were.

The amendment as given below has some good points, especially, so far as it refers to dental college requirements; in this particular it is an improvement on the law that was enacted two years ago. The chief aim of this amendment, so far as it appears on its face, is to define what constitutes a reputable dental college. This, under the former law, devolved upon the State Board of Examiners; this amendment relieves the Board of this responsibility. It can not in any wise affect any graduates who go from Ohio to any other State to practice.

If the State Board is careful to fully secure full and complete knowledge of the various colleges whose graduates present themselves for registration, this amendment will be helpful rather than otherwise. This enactment will stimulate to closer scrutiny, especially of the colleges whose methods are not fully known.

SECTION I —*Be it enacted by the General Assembly of the State of Ohio*, That Sec. 4,404 of Revised Statutes, as amended April 8, 1892 (87 O. S. L. p. 237), be supplemented as follows, with sectional numbering 4,404 A :

SEC. 4,404, That for the purposes of this Act, Colleges of Dentistry shall be regarded as reputable which are under State control, or are organized, controlled and governed by a Board of Trustees, as provided by law for governing colleges of medicine, which possess buildings, by lease or otherwise, and equipments valued at not less than five thousand dollars, which have a graded course of not less than three years, the time of instruction in each year being not less than six months, and which have a curriculum which includes anatomy, physiology, histology, pathology, chemistry, microscopy, materia medica, metallurgy, operative, mechanical and surgical dentistry.

SEC. II.—This act shall take effect on and after its passage.

ALEX. BOXWELL,
Speaker of the House of Representatives.
ANDREW L. HARRIS,
President of the Senate.

Passed May 21st, 1894.

